# Utilization of iron–folic acid supplementation and related factors in pregnant women in Leka Dulecha District, East Wollega Zone, Western Ethiopia: The case study

**DOI:** 10.1002/hsr2.1952

**Published:** 2024-03-12

**Authors:** Chimdessa Tolera, Temesgen Tafesse, Ra'el Dessalegn, Desalegn Amenu

**Affiliations:** ^1^ Department of Public Health, Institute of Health Sciences Wollega University Nekemte Ethiopia; ^2^ Armauer Hansen Research Institute Malaria and Neglected Tropical Diseases Addis Ababa Ethiopia; ^3^ Department of Biology, College of Natural and Computational Science Wollega University Nekemte Ethiopia

**Keywords:** infants and pregnant women, iron–folic acid utilization, Ministry of Health

## Abstract

**Background:**

To treat neural tube closure abnormalities and maternal anemia during pregnancy, iron and folic acid (FA) supplements are typically necessary. Ethiopian Ministry of Health plan to increase the numbers of pregnant women who take iron and FA supplements from 11% to 50%, and by 2029, to 90% by 2024.

**Aim:**

Hence, the main objective of this study was to investigate the degree of iron–folic acid supplementation (IFAS) and associated factors among pregnant women receiving antenatal care at Leka Dulecha Woreda public health facilities from May 1 to October 31, 2022.

**Methods:**

In this study, about 316 pregnant women who visited Leka Dulecha prenatal care services were selected. A facility‐based cross‐sectional study was conducted. Multivariable logistic regression was utilized to examine parameters associated with the utilization of IFAS.

**Results:**

These findings suggest that maternal educational status (adjusted odds ratio, AOR = 2.00, 95% confidence interval, CI [1.5, 3.05]), the timing of the first prenatal consultation (AOR = 1.93, 95% CI [1.47, 2.62]), having a good understanding of anemia (AOR = 1.50, 95% CI [1.00, 2.11]), and a history of anemia during the current pregnancy (AOR = 1.60, 95% CI [1.11, 3.16]) are important factors to consider when promoting adherence to iron–FA supplementation among pregnant women.

**Conclusion:**

It is crucial for healthcare providers to address these factors to improve the overall health outcomes for pregnant women attending Leka Delecha Health Facility.

## 1 INTRODUCTION

Iron–folic acid (FA) deficiency anemia is a worldwide public health issue, particularly in low‐ and middle‐income nations such as Ethiopia. This state is critical for the mother's physiological function, growth, and development throughout pregnancy and in later years. Pregnancy raises the requirement for iron and FA, influencing the fetus' growth and development.^1^ Adherence to iron–folic acid supplementation (IFAS) throughout pregnancy may benefit both the mother and the fetus. Increased adherence lowers the risk of anemia, hemorrhagic infant illness, and congenital defects, making iron deficiency anemia the most prevalent hematological problem in pregnant women and children.^1^, ^2^, ^3^

Pregnancy‐related adverse birth outcomes, including neural tube defects, cardiac defects, and endocrine disorders, are linked to low iron–FA (IFA) intake. Supplementation is recommended to prevent these complications.^4^, ^5^ Adhering to a higher IFA regimen during pregnancy leads to increased productivity and a reduced risk of iron deficiency anemia, bleeding, sepsis, and maternal death.^6^ Conversely, low adherence can impair immune function, vitality, and brain development and can result in low birth weight, preterm birth, and postnatal complications. It is important for pregnant women to prioritize their health and follow the recommended IFA regimen to ensure a healthy pregnancy and positive outcomes for both mother and baby.^7^

According to the 2016 Ethiopian Demographic and Health Survey, 23% of Ethiopian women between the ages of 15 and 49 are anemic, of which 15% are somewhat, 5% are extremely, and less than 1% are severely. In addition, a great deal of effort has been made to reduce the global anemia prevalence by the end of 2025. Therefore, the purpose of this study was to determine the factors influencing supplementation as well as IFAS among pregnant mothers in Leka Dulecha Woreda, East Wollega Zone, Western Ethiopia.

## 2 MATERIALS AND METHODS

### 2.1 Study area and period

**Figure 1:**
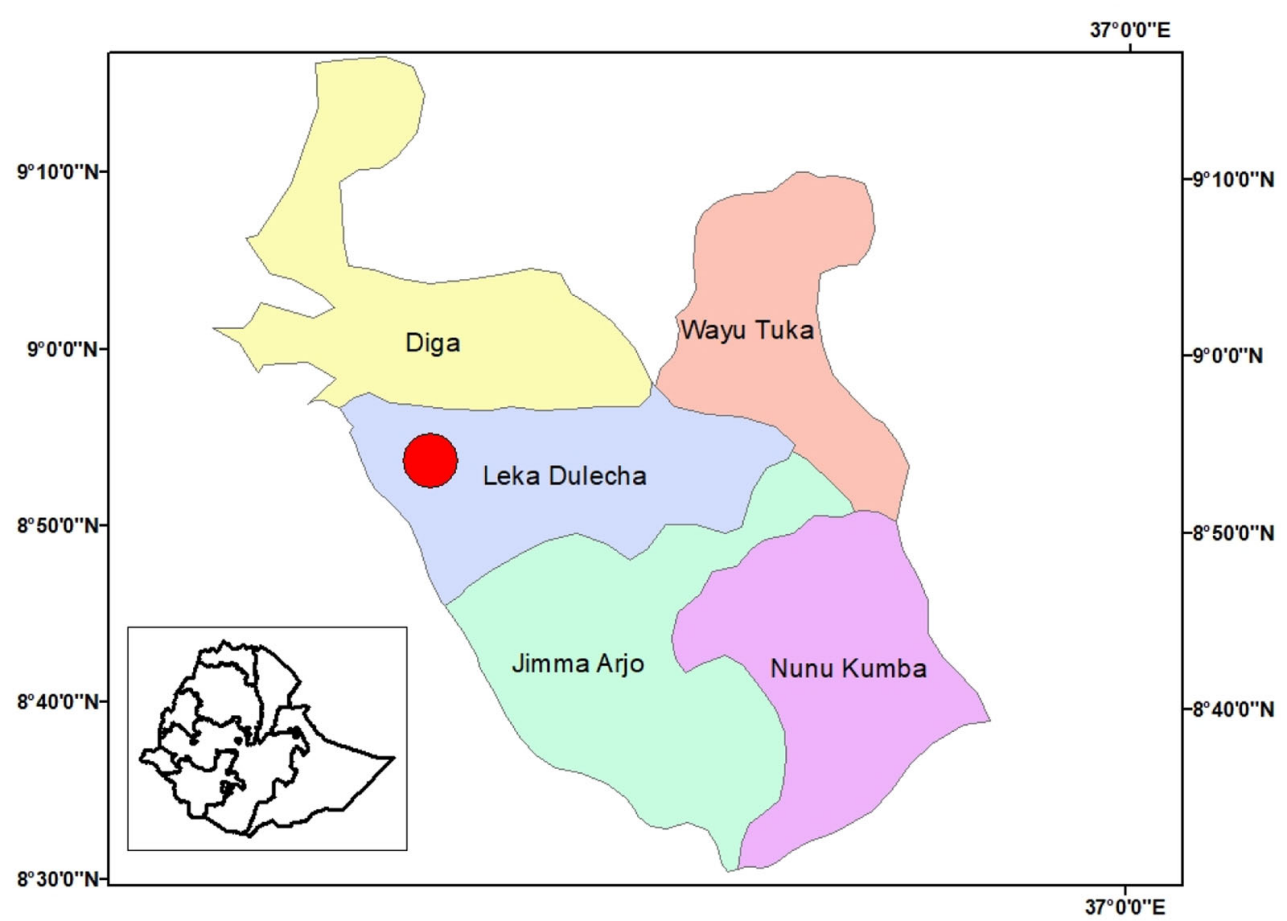
Map of the study area, East Wollega Zone (Leka Dulecha District).

The study's focus was on public health facilities in the East Wollega Zone of Leka Dulecha Woreda, which has 105,412 residents, 169 medical professionals, and 31 support staff. With 4 health facilities, 22 rural and 2 urban health posts, and comprehensive preventative, promotional, curative, and rehabilitative health services, the Woreda Public Health Institution serves the community (Figure 1).

### 2.2 Study design

An investigation was conducted at the Leka Dulecha Health Center, located in the Leka Dulecha District of the East Wollega Zone of the Oromia Regional state, using a cross‐sectional approach, from May 1 to October 30, 2022.

#### 2.2.1 Source and study population

Pregnant women who received antenatal care (ANC) services at public health facilities Leka Dulecha Woreda comprised the source population for this research.

#### 2.2.2 Inclusion and exclusion criteria

ANC visits made at least 3 months prior were included in the sample; pregnant women who visited Leka Dulecha Woreda health facilities frequently and those who were not mentally or physically capable of conducting interviews were excluded.

#### 2.2.3 Sample size and sampling techniques

The sample size was calculated using a single population proportion formula using a 95% confidence level, a 5% margin of error, a prevalence of 11.5%wt,^8^ a design effect of 1.5, and a nonresponse rate of 10%. The first volunteer was selected at random by lottery, and the second was involved in each *K* value (i.e., *K* = 2) for all healthcare facilities. As a result, a single population formula was utilized. Of the 316 samples that were collected were Getema Health Center, Bandira Health Center, Horda Ambalta Health Center, and St. Dan. Combon Health Center received 134, 92, 51, and 39 samples, respectively. As a result, just one population formula was employed; the first volunteer was selected at random by lottery, and the second was included in each *K* value (*K* = 2) for all medical facilities. From a total of 316 samples, Getema Health Center, Bandira Health Center, Horda Ambalta Health Center, and St. Dan. Combon Health Center received 134, 92, 51, and 39 samples, respectively.

### 2.3 Data collection tools and procedure

A structured questionnaire that was adapted from a review of pertinent literature^9^, ^10^ was used to conduct face‐to‐face interviews.^11^ Supervisors and data collectors received extensive training on the goals of the study, how to manage questionnaires, how to collect data, and how to evaluate ethical issues. Six female enumerators who had received training and were fluent in the local language were used to collect the data. They had also previously participated in data collection. Four BSc health experts assisted in overseeing the process of gathering data. Supervisors exercised strict monitoring, while study investigators kept an eye on the general caliber of data collection. Every day, the supervisors received the data that the enumerators had gathered. The supervisors received the data that the enumerators had gathered and ensured its accuracy and completeness.

### 2.4 Data analysis

A binary logistic regression model was employed in the study to examine the association between independent variables and the current use of IFAS by pregnant women. The multivariable logistic regression model contained variables with *p* values less than 0.25. Model fitness was evaluated using Hosmer and Lemeshow's goodness of fit test, while multicollinearity was evaluated using variance inflation factors. After calculating the adjusted odds ratio (AOR), the findings were shown in text, tables, and graphs.

### 2.5 Data quality assurance

After examining several works of literature on the subject, the questionnaires are modified and translated into Afan Oromo.^9^, ^10^, ^11^ Data collectors verified the consistency and completeness of the questionnaires on a daily basis while gathering data. Lastly, before the questionnaire was input into the computer program and examined by the principal investigator, its completeness was confirmed. Epidata version 3.1 was finally used to finish the data entry process.

### 2.6 Ethical clearance

Before the start of data collection and pretest, an ethical clearance letter was obtained from the Leka Dulecha and Arjo Woreda H/Office and sent to the Administrative Office of Governmental Health Facilities in Leka Dulecha, Woreda, and Arjo health centers, along with the reference number (SMidw/125/2022). Participants in the study were given the assurance that the information collected would be kept confidential during data collection.

#### 2.6.1 Study variables

The compliance with iron and FA supplementation was the dependent variable. The following were the independent variables: Sociodemographic and economic traits, traits connected to maternal health services, traits related to obstetrics, and awareness of IFA supplementation and anemia.

#### 2.6.2 Operation definitions

Questions concerning the causes, symptoms, prevention, and treatment of anemia were used to gauge participants' knowledge of the condition. IFA‐recommended questions on the uses of IFA pills, how long is an appropriate intake, when to start taking supplements, and adverse effects were used to gauge the degree of knowledge. One point was awarded for a correct response, and zero for a bad one. A high understanding of anemia and IFA was determined by properly answering more than 80% of the questions in the knowledge assessment. Furthermore, mothers were classified as having medium and poor knowledge of anemia and the IFA, respectively, if they had correctly answered 60%–80% and less than 60% of the knowledge assessment questions.

## 3 RESULTS

### 3.1 Sociodemographic description of the study participants

The study participants' average age was 28.62 years, with a 5.6‐year standard deviation. A total of 176 (56.6%) of the responding expecting mothers were between the ages of 25 and 34, while 77 (24.8%) were younger than 24. In terms of the expecting mothers' educational standing, roughly 54 (17.4%) of the study participants had completed at least a primary education, whereas 95 (30.5%) of the pregnant women had completed at least a primary education. The bulk of the respondents were from metropolitan regions, while the pregnant women were primarily from rural areas (Table 1).

**Table 1:** The sociodemographic features of expectant mothers receiving ANC at Leka Dulecha Woreda Health Facilities between May 2022 and October 2022.

Variable	Coding category	Frequency (*n*)	Percentage (%)
Age of the respondents	≤24 years	77	24.8
	25–34 years	176	56.6
	**≥**35 years	58	18.6
Total		311	100
Educational status	Cannot read and write	139	44.7
	Read and write	23	7.4
	Primary education	95	30.5
	Secondary education and above	54	17.4
	Total	311	100
Occupation status	Salaried employee	44	14.1
	Farmer	162	52.1
	Self‐employment/business	49	15.8
	Housewife	44	14.1
	Daily laborer	12	3.9
	Total	311	100
Area of residence	Urban	75	23.9
	Rural	239	76.1
	Total	311	100
Marital status	Married	296	95.2
	Other	15	4.8
	Total	311	100
Ethnicity	Oromo	276	88.7
	Other	35	11.3
	Total	311	100
Religion	Orthodox	118	37.9
	Protestant	177	56.9
	Other	16	5.1
	Total	311	100
Spousal education	Cannot read and write	109	35.0
	Write and read	30	9.6
	Primary education	98	31.5
	Secondary education and above	74	23.8
	Total	311	100

Abbreviation: ANC, antenatal care.

### 3.2 Description of obstetrics‐related factors of the study participants

**Figure 2:**
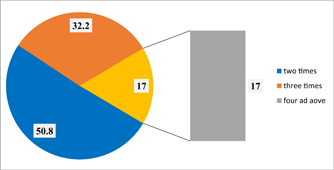
Frequency of ANC visits, among pregnant women attending ANC at Leka Dulecha Woreda Health Facilities, May 1–30, 2022. ANC, antenatal care.

At the time of the interview, 200 (64.3%) of the expecting moms were in their third trimester of pregnancy. The remaining women were in the first or second trimester of pregnancy. The majority of pregnant moms were first‐time mothers, making up 279 (89.7%) of the respondents who were multigravida or pregnant for the second time or beyond. The highest parity number was 8, and the mean delivery was 2.94 with a standard deviation of ±2.103. 212 (68.2%) out of the total responses were multiparous. Hence, only 41 pregnant women (13.2%) had given birth to a stillborn child at least once in their prior pregnancies. Furthermore, out of all respondents, 52 (16.7%) of the women had already had an abortion at some point in their lives (Figure 2).

A total of 134 pregnant women (43.1%) started ANC follow‐up in the second trimester of their pregnancy, while 132 respondents (42.4%) had their first ANC visit in the first trimester and the remaining respondents in the third. A health center accounted for 248 out of the total respondents, or 79.7%, of whom had their first ANC visit; the other respondents had their first ANC visit at a hospital or health post (Table 2).

**Table 2:** Obstetric‐related factors of pregnant women attending ANC at Leka Dulecha Woreda Health Facilities, May 1–30, 2022.

Variable	Coding category	Frequency (*n*)	Percentage (%)
Gestational age of the mothers	First trimester	19	6.1
	Second trimester	92	29.6
	Third trimester	200	64.3
	Total	311	100
Number of pregnancy	Primigravida	32	10.3
	Multigravida	279	89.7
	Total	311	100
Number of delivery	Nulliparous	37	11.9
	Primipara	62	19.9
	Multipara	212	68.2
	Total	311	100
Frequency of ANC visit	Two times	158	50.8
	Three times	100	32.2
	Four times	53	17.0
	Total	311	100
Place of ANC visit	health post	30	9.6
	health center	248	79.7
	hospital	33	10.6
	Total	311	100
Gestational age at first visit of ANC	first trimester	132	42.4
	second trimester	134	43.1
	third trimester	45	14.5
	Total	311	100
History of still birth	yes	41	13.2
	no	270	86.8
	Total	311	100
History of abortion	Yes	52	16.7
	No	259	83.3
	Total	311	100

Abbreviation: ANC, antenatal care.

### 3.3 The knowledge status about anemia and IFA supplement

#### 3.3.1 Knowledge status of respondents about anemia

**Figure 3:**
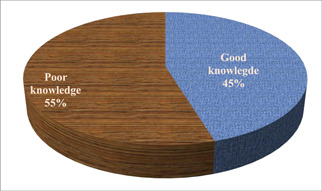
The knowledge status about anemia, among pregnant women attending ANC at Leka Dulecha Woreda Health Facilities, May 1–30, 2022. ANC, antenatal care.

Using anemia knowledge‐related questions about signs and symptoms, causes, complications, preventive measures, and other topics, the respondents’ level of anemia knowledge was assessed. The respondent's level of awareness about anemia was categorized using the mean value. 11.05. was the average. Pregnant women with scores above the mean were considered to have strong knowledge of anemia, whereas those with scores below the mean were thought to have weak knowledge. Consequently, 170 (54.7%) of the participants knew very little about anemia, whereas 141 (45.3%) knew a lot about it (Figure 3).

#### 3.3.2 Knowledge status of respondents about IFAS

**Figure 4:**
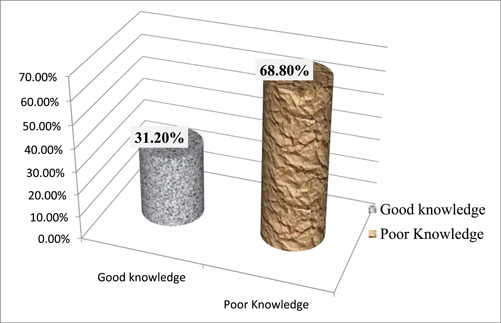
The knowledge status about IFAS, among pregnant women attending ANC at Leka Dulecha Woreda Health Facilities, May 1–30, 2022. ANC, antenatal care; IFAS, iron and folic acid supplementation.

Using nine IFAS‐related knowledge questions on when to take it, the consequences of not taking it, the significance of taking it regularly, and other issues, the respondents’ understanding of IFA supplements was also evaluated. 4.37 was the average. Women who were expecting and had scores above the mean were thought to have a good understanding of IFAS, whereas those who had scores below the mean were thought to have poor knowledge. As a consequence, 97 (31.2%) of the participants had a strong grasp of IFAS, compared to 214 (68.8%) who had a weak understanding (Figure 4).

#### 3.3.3 Description of health facility‐related factors

The study participant's assessment of the factors related to health facilities was based on four pertinent variables: the participant's distance from the facility, advice on the significance of IFAS during pregnancy, the participant's experience during their ANC visit, and the participant's close follow‐up by the healthcare provider. The time it took for residents to get to the health facility was used to estimate the distance. Of the 145 respondents, nearly half (46.6%) took between 31 and 60 min to get there, while about 87 respondents (or 28% of the sample) took 61 min or longer, and the remaining respondents took 30 min or less.

During their ANC visit, 185 out of the respondents (59.5%) received advice or information about the importance of IFAS; however, 126 out of the respondents (40.5%) did not receive this guidance. During their ANC visit for the current pregnancy, nearly half of the respondents, 153 (49.2%), experienced issues related to the health facility, such as a lack of supplements in the facility 48 (15.4%), a lengthy wait time for service 24 (7.7%), and inadequate communication from the healthcare provider during the ANC visit 22 (7.1%) (Table 3).

**Table 3:** Health facility‐related factors of pregnant women attending ANC at Leka Dulecha Woreda Health Facilities, May 1–30, 2022.

Variable	Coding category	Frequency (*n*)	Percentage (%)
Traveling time to the health facility	Less than 30 min	79	25.4
	31–60 min	145	46.6
	More than 61 min	87	28.0
Did HW talk about the importance of IFAS	Yes	185	59.5
	No	126	40.5
Face problem during ANC visit	Yes	153	49.2
	No	158	50.8
Follow up by health care provider	Yes	191	61.4
	No	120	38.6

Abbreviations: ANC, antenatal care; IFAS, iron and folic acid supplementation.

Of the 191 research participants, 16% (61.4%) had their usage of IFAS followed up on, either by setting up an appointment and counting pills, or by putting expectant mothers in touch with the women's development army or health extension workers in the relevant kebele (Table 3).

### 3.4 IFA supplement utilization

**Figure 5:**
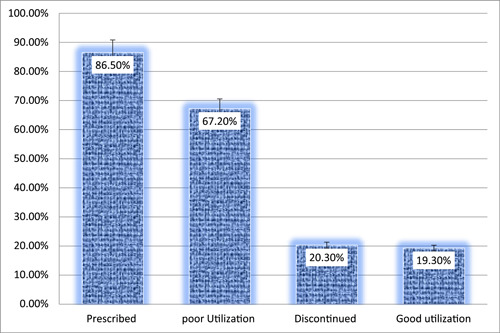
IFAS status, among pregnant women attending ANC at Leka Dulecha Woreda Health Facilities, May 1–30, 2022. ANC, antenatal care; IFAS, iron and folic acid supplementation.

The study's conclusions show that 86.5 percent of the 269 respondents received and used iron and FA supplements at least once throughout their current pregnancy. Only 38 (14%) of the mothers‐to‐be started taking iron and FA supplements in the first 3 months of their pregnancies, while 232 (86%) started in the fourth month. The average age at which research participants began taking iron and folic supplements was 4.38 months of pregnancy. Women who took iron and FA supplements throughout a recent pregnancy did so for a duration ranging from 20 to 120 days. The average duration of IFAS for the study participant was 60 days, with a standard deviation of 28.18. Only one‐fifth of the trial participants (19.3%) took and consumed the IFA supplement of 90 tablets or more (Figure 5).

**Figure 6:**
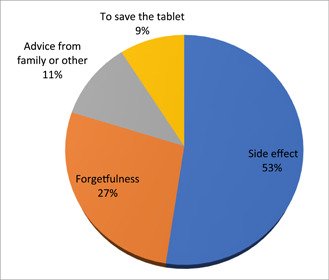
The reason why not consume IFAS on a daily basis, among pregnant women attending ANC at Leka Dulecha Woreda Health Facilities, May 1–30, 2022. ANC, antenatal care; IFAS, iron and folic acid supplementation.

Of the women who had taken IFA supplements, 234 (86.9%) came from the health center, with the remaining women receiving their supplements from the hospital and health post. About 208 (77.3%) of the pregnant women who took IFAS did not take the supplement every day. The most common reason (109, or 40.5%) for not taking the IFA supplement on a daily basis was side effects from the pill; forgetfulness accounted for 57 cases (21.2%), followed by family members' recommendations (23, or 8.5%), and saving the tablet was the remaining reason (Figure 6).

**Figure 7:**
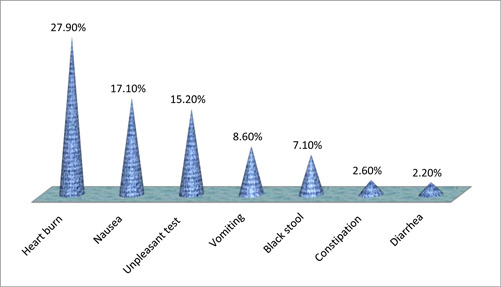
Experienced side effects of the supplement, among pregnant women attending ANC at Leka Dulecha Woreda Health Facilities, May 1–30, 2022. ANC, antenatal care.

About 162 (60.2%) of the pregnant women who took iron and FA supplements reported experiencing negative side effects. According to the study's findings, heartburn accounted for 75 (27.9%) of all adverse effects. Other common side effects included nausea (46 [17.1%]), bad taste (41 [15.2%]), vomiting (23 [8.6%]), black stool (7.1%), constipation (2.60%), and diarrhea (2.2%) (Figure 7).

**Figure 8:**
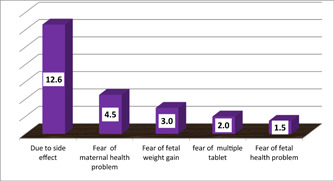
The reason why not consume IFAS on a daily basis, among pregnant women attending ANC at Leka Dulecha Woreda Health Facilities, May 1–30, 2022. ANC, antenatal care; IFAS, iron and folic acid supplementation.

Of those who had received a prescription and begun taking the supplement, 63 (23.4%) stopped taking it before the allotted 90 days had passed. Side effects accounted for 34.6% of the discontinuation cases, followed by maternal health issues (12.5%), intrauterine fetal weight gain (8.3%), taking too many tablets (5.2%), and fetal health issues (4.5%) (Figure 8).

Entirely, 42 (13.5%) of the total respondents who were pregnant did not receive IFA supplements during their recent pregnancy. The lack of iron tablets in the medical facility at the time of the visit 25 (60.97%), lack of knowledge about the significance of IFAS 6 (14.6%), confusion about where to obtain the supplement 5 (12.19%), and forgetting to take the supplement 5 (12.19%) were the reasons given for not taking the IFA supplement during the recent pregnancy.

### 3.5 Factors associated with IFAS utilization among the study participants

The study found that a number of characteristics, including maternal education, the timing of ANC registration, the history of anemia, the prevalence of anemia during the current pregnancy, IFA counseling, and knowledge of IFAS and anemia, were associated with adherence to IFAS. The multivariate logistic regression analysis revealed significant relationships between IFAS adherence and the timing of ANC registration, the prevalence of anemia in the current pregnancy, maternal education, awareness of anemia, and IFAS knowledge. These results imply that encouraging adherence to IFAS requires early ANC registration and a sufficient understanding of anemia and IFAS. It also emphasizes how crucial it is to raise pregnant women's knowledge of anemia to increase their adherence to.

The likelihood of pregnant women sticking to IFAS was twice as high for those who completed secondary school or above as it was for those who are illiterate (AOR = 2.00, 95% CI [1.5–3.05]). Early ANC enrollment by pregnant women was associated with a 1.93‐fold higher chance of sticking to IFAS than later enrollment (AOR = 1.93, 95% CI [1.47–2.62]). The likelihood of pregnant women adhering to IFAS was 1.5 times higher for those who knew about anemia than for those who didn't (AOR = 1.50, 95% CI [1.00, 2.11]). When a pregnant woman knew about IFAS, her chances of following them were 1.25 times higher than when she didn't know about them (AOR = 1.25, 95% CI [1.14–2.34]). Compared to women without anemia, those with a history of anemia during their current pregnancy were 1.60 times more likely to follow IFAS guidelines (AOR = 1.60, 95% CI [1.11, 3.16]) (Table 4).

**Table 4:** Factors associated with iron–folic acid supplement utilization among pregnant women attending ANC service at Leka Dulecha Woreda Public Health Facilities, May 1–30, 2022.

Variables	Adherences of IFAS			
Educational status	Yes	No	COR (95% CI)	AOR (95% CI)
Can't read and write	12	74	1	1
Read and write only	14	64	1.35 (1.05–2.20)	1.29 (1.01–1.24)**
Primary education	21	51	1.89 (1.11–2.75)	1.75 9 (1.13–1.40)**
Secondary and above	40	35	2.75 (2.09–2.82)	2.00 (1.5–3.05)**
Marital status				
Married	150	100	3.03 (2.33–3.75)	1.10 (1.40–1.80)
Unmarried	50	11	1	1
Time of ANC registration				
Early, <12 week	95	37	2.85 (2.26–3.41)	1.93 (1.47–2.62)**
Late, >12 week	175	24	1	1
Knowledge of anemia				
Knowledgeable	80	50	4.75 (4.10–5.40)	1.50 (1.00–2.11)**
Less knowledgeable	160	21	1	1
Knowledge of IFAS				
Knowledgeable	128	42	2.10 (1.50–2.70)	1.25 (1.14–2.34)**
Less knowledgeable	122	19	1	1
Counseling on IFAS				
Yes	25	70	1.80 (1.27–1.89)	1.58 (1.05–2.12)
No	85	132	1	1
History of anemia				
Yes	26	75	2.00 (1.40–2.41)	1.60 (1.11–3.16)
No	84	128	1	1

Abbreviations: ANC, antenatal care; AOR, adjusted odds ratio; CI, confidence interval; COR, crude odds ratio; IFAS, iron and folic acid supplementation.

** Variables associated in multivariate analysis and statistically significant at *p* < 0.05.

## 4 DISCUSSION

Finding out how much IFAS there was among pregnant women obtaining ANC services at public health institutions in Leak Dulecha woreda, as well as the factors linked to this utilization, was the aim of the study. 311 of the 316 pregnant women who were part of the study answered the questionnaires, yielding a 98.4% response rate overall.

Nearly, 60% of pregnant women received more than 90 tabs of the recommended IFA supplements tablets out of all the pregnant women receiving ANC services at Leka Dulecha Woreda public health facilities during the study period. A 19.3% utilization rate is the result of this. This outcome is consistent with research from studies carried out in Kasulu, North West Tanzania, and Amhara, Northern Ethiopia, which found that the rates of IFA consumption were 20.4% and 20.3%, respectively.^12^, ^13^

Nonetheless, this figure is greater than the 11% from the EDHS 2019, the 11.5% from a different study done in Wolaita, Southern Ethiopia, the 3.5% from eight rural districts in Ethiopia, and the 17.4% from a study done in a high‐focus state in India.^8^, ^14^, ^15^, ^16^ This discrepancy could be caused by the current study's temporal fluctuation, study scale, study site variance, and differences in sociodemographic traits. Another explanation for the observed difference could have to do with the clients' increased awareness of the value of utilizing IFA, which has been made possible by acting partners in the program, particularly in the district.

According to Ba et al.,^17^ Jenberu,^18^ Kamau et al.,^19^ Titilayo et al.,^20^ and other studies conducted in Sub‐Saharan countries, the IFA consumption rate found in this study is lower than that of previous studies conducted in Kirkos, Addis Ababa, Ethiopia (28.1%), Kimbu County, Kenya (32.7%), Malawi (37%), Nepal (23%), and other countries. The fact that the city in the previous study was far more urbanized than the city in this study could account for the difference in the amount of IFAS consumption. The disparity between the two nations could be attributed to sociocultural variations in the study groups, study scope, and study location.

The variables associated with the use of IFA supplements were discussed by research participants. Four variables were determined to be statistically significant in the final model. The degree of education of the respondent, the frequency of ANC visits, the knowledge of anemia, and the side effects of medications are these variables.

According to this study, expecting mothers who had completed their primary education or more had a nearly fourfold higher likelihood of using iron and FA supplements than those who had not. This finding is in line with research conducted in the Amara region of Ethiopia (AOR: 4.49, 95% CI: 2.61–7.76) and a high‐focus state of India (OR = 4.21; 95% CI = 3.30–5.36), both of which found that the respondent's education level had a significant association with consumption of iron folic supplements. This is because women who have completed formal education are four times more likely to use iron.^13^, ^14^ This may be because a woman's educational background influences her ability to weigh the pros and cons of taking FA and iron supplements while pregnant. Moreover, mothers with greater education may encounter a multitude of resources endorsing the advantages of supplementing with iron and FA.

The frequency of ANC visits was substantially correlated with the usage of iron and FA supplements. ANC is an essential way to replenish iron and FA during pregnancy. Consequently, the expected relationship between the frequency of ANC services and the usage of iron and FA supplements has been found. AOR = 1.93, 95% CI (1.47–2.62) showed that pregnant mothers who attended ANC were twice as likely to use IFAS of 90 tabs as those who attended ANC less frequently. This outcome is in line with research conducted in Dire Dawa, eastern Ethiopia (AOR = 3.15, 95% CI: 1.16–9.55), the Philippines DHS 2017 (AOR: 2.71, 95% CI: 2.08–3.52), and another study on IFAS and its relationship to the number of ANC visits in Ethiopia (AOR = 2.54; 95% CI: 1.43–4.50).

Pregnant women who have three or more ANC visits are more likely to use iron and FA, according to the findings of many research conducted in various regions.^21^, ^22^ The reason for this could be that health providers could help women at their frequent ANC visits by educating them about the health benefits of taking an iron and FA supplement for both mothers and the unborn child, encouraging them to take the tablet as directed, and discussing the significance of consuming an iron and FA supplement during pregnancy.

Pregnant women who knew they had anemia were 1.5 times more likely to follow IFAS in this study than those who didn't know (AOR = 1.50, 95% CI (1.00, 2.11)). This result is in line with studies from the Amara region of Ethiopia (AOR: 3.64, 95%CI: 1.78–7.39) and Kasulu, northwest Tanzania (AOR = 3.840, 95% CI: 1.335, 10.685), which found that pregnant women who knew a lot about anemia were four times more likely to use IFA 90 tabs or more.^12^, ^13^

Furthermore, even though the results differ from this study's, other studies—such as those carried out in the Gulale sub‐city of Addis Ababa, Ethiopia (AOR = 1.97; 95% CI = 1.24–3.13), Walaita, South Ethiopia (AOR = 0.15; 0.04–0.62), and eight rural districts of Ethiopia (OR = 0.75; 95% CI: 0.57)^8^, ^16^, ^23^, ^24^ all demonstrate a statistically significant correlation between anemia knowledge and the use of IFAS. This important variable may be explained by the fact that pregnant mothers who are aware of the iron deficiency anemia's causes, symptoms, effects, and preventative strategies take IFA supplements because they are concerned about the anemia's potential repercussions on both the mother and the fetus.

In contrast to the national food and nutrition strategy, which considered consuming IFAS at least 90 tabs as an outcome indicator for measuring IFAS during pregnancy, and the Ethiopian national nutrition program recommendation, which aimed to increase the proportion of pregnant women who receive IFA, it was discovered that the use of IFA supplements was low among pregnant women attending antenatal care at Leka Dulecha Woreda health facilities. advising expectant mothers to take IFA supplements at any point during IFA pill collection or at subsequent ANC visits, with an emphasis on the benefits of doing so, any possible drawbacks, and strategies for mitigating such effects.

## AUTHOR CONTRIBUTIONS

**Chimdessa Tolera**: Conceptualization; investigation; funding acquisition; writing—original draft; methodology; validation; visualization; writing—review and editing; software; formal analysis; project administration; data curation; supervision; resources. **Temesgen Tafesse**: Data curation; software; conceptualization; investigation; funding acquisition; writing—original draft; methodology; validation; visualization; writing—review and editing; project administration; formal analysis; supervision; resources. **Ra'el Dessalegn**: Conceptualization; investigation; funding acquisition; writing—original draft; methodology; validation; visualization; writing—review and editing; project administration; formal analysis; software; data curation; supervision; resources. **Desalegn Amenu**: Conceptualization; investigation; funding acquisition; writing—original draft; methodology; validation; visualization; writing—review and editing; project administration; formal analysis; software; data curation; supervision; resources. all authors have read and approved the final version of the manuscript.

## CONFLICT OF INTEREST STATEMENT

The authors declare no conflict of interest.

## ETHICS STATEMENT

An ethical letter, bearing reference number (SMidw/125)2022, was obtained from the East Wollega Zone's Health Office in Leka Dulecha and Arjo. It was then delivered to the Administrative Office of Governmental Health Facilities in Leka Dulecha, Woreda, and Arjo. There was also a permit document from the Leka Dullecha district health office.

## TRANSPARENCY STATEMENT

The lead author Desalegn Amenu affirms that this manuscript is an honest, accurate, and transparent account of the study being reported; that no important aspects of the study have been omitted; and that any discrepancies from the study as planned (and, if relevant, registered) have been explained.

## Data Availability

All data used in this study were included in the manuscript, table, and figures; there are no any data or supplementary data. Desalegn Amenu had full access to all of the data in this study and takes complete responsibility for the integrity of the data and the accuracy of the data analysis.
